# Has the COVID-19 pandemic changed existing patterns of non-COVID-19 health care utilization? A retrospective analysis of six regions in Europe

**DOI:** 10.1093/eurpub/ckad180

**Published:** 2024-07-01

**Authors:** Sarah J Aldridge, Andrea E Schmidt, Martin Thißen, Enrique Bernal-Delgado, Francisco Estupiñán-Romero, Javier González-Galindo, Lorenz Dolanski-Aghamanoukjan, Stefan Mathis-Edenhofer, Tamara Buble, Klea Križ, Jakov Vuković, Luigi Palmieri, Brigid Unim, Iris Meulman, Rhiannon K Owen, Ronan A Lyons

**Affiliations:** Population Data Science, Swansea University Medical School, Faculty of Medicine, Health, and Life Science, Swansea University, Swansea, UK; Competence Centre Climate and Health, GÖG (Austrian National Public Health Institute), Vienna, Austria; Department for Health Monitoring and Epidemiology, Robert Koch Institute, Berlin, Germany; Data Science for Health Services and Policy Research Group, Institute for Health Sciences in Aragon (IACS), Zaragoza, Spain; Data Science for Health Services and Policy Research Group, Institute for Health Sciences in Aragon (IACS), Zaragoza, Spain; Data Science for Health Services and Policy Research Group, Institute for Health Sciences in Aragon (IACS), Zaragoza, Spain; International Affairs, Policy, Evaluation, Digitalisation, GÖG (Austrian National Public Health Institute), Vienna, Austria; Health Care Planning and System Development, GÖG (Austrian National Public Health Institute), Vienna, Austria; Croatian Institute of Public Health (HZJZ), Zagreb, Croatia; Croatian Institute of Public Health (HZJZ), Zagreb, Croatia; Croatian Institute of Public Health (HZJZ), Zagreb, Croatia; Department of Cardiovascular, Endocrine-Metabolic Diseases and Aging, Istituto Superiore di Sanità (ISS), Rome, Italy; Department of Cardiovascular, Endocrine-Metabolic Diseases and Aging, Istituto Superiore di Sanità (ISS), Rome, Italy; Center for Public Health, Health Services and Society, National Institute for Public Health and the Environment, Bilthoven, The Netherlands; Tranzo, Tilburg School of Social and Behavioural Sciences, Tilburg University, Tilburg, The Netherlands; Population Data Science, Swansea University Medical School, Faculty of Medicine, Health, and Life Science, Swansea University, Swansea, UK; Population Data Science, Swansea University Medical School, Faculty of Medicine, Health, and Life Science, Swansea University, Swansea, UK

## Abstract

**Background:**

Resilience of national health systems in Europe remains a major concern in times of multiple crises and as more evidence is emerging relating to the indirect effects of the COVID-19 pandemic on health care utilization (HCU), resulting from de-prioritization of regular, non-pandemic healthcare services. Most extant studies focus on regional, disease specific or early pandemic HCU creating difficulties in comparing across multiple countries. We provide a comparatively broad definition of HCU across multiple countries, with potential to expand across regions and timeframes.

**Methods:**

Using a cross-country federated research infrastructure (FRI), we examined HCU for acute cardiovascular events, elective surgeries and serious trauma. Aggregated data were used in forecast modelling to identify changes from predicted European age-standardized counts via fitted regressions (2017–19), compared against post-pandemic data.

**Results:**

We found that elective surgeries were most affected, universally falling below predicted levels in 2020. For cardiovascular HCU, we found lower-than-expected cases in every region for heart attacks and displayed large sex differences. Serious trauma was the least impacted by the COVID-19 pandemic.

**Conclusion:**

The strength of this study comes from the use of the European Population Health Information Research Infrastructure’s (PHIRI) FRI, allowing for rapid analysis of regional differences to assess indirect impacts of events such as pandemics. There are marked differences in the capacity of services to return to normal in terms of elective surgery; additionally, we found considerable differences between men and women which requires further research on potential sex or gender patterns of HCU during crises.

## Introduction

Resilience of national health systems in Europe remains a major concern in times of multiple crises, even as the Coronavirus Disease 2019 (COVID-19) pandemic has been declared to no longer be a public health emergency of international concern as of 5 May 2023.^1^^,^^2^ Slowly, more evidence is emerging as to the indirect effects of the pandemic on health care utilization and the pandemic’s potential mid-term effects resulting from postponed treatments and a de-prioritization of regular, non-pandemic healthcare services both for elective treatments and emergency care,^3^^,^^4^ given the shock and unprecedented impact that European healthcare systems experienced from February 2020 onwards,^1^^,^^5^ and the consequent redirecting of resources to the pandemic response. In fact, some strategies taken to tackle rising COVID-19 infection rates, such as advising the public to avoid all but essential contact with the health service and stopping elective activities, are likely to have negatively affected access to health services for other conditions. Yet, extant studies are hardly comparable with each other and often focus on analysing the impact on the use of services for specific diseases or population groups during the first stages of the pandemic,^3^ with a few exceptions (see e.g. Ref. [6]) Also, few studies have looked at health care utilization (HCU) patterns separately for men and women.^3^

Furthermore, while most studies focus on one specific condition,^5–9^ our study differentiates between three different types of conditions, with differing implications for health systems capacity during the pandemic: hospital admissions for acute cardiovascular events (represented by heart attack and stroke diagnosis), elective major joint replacement surgery (arthroplasty) and serious trauma as defined by Cryer et al.^10^ Firstly, the impact of COVID-19 on cardiovascular disease has raised substantial research interest, with results from numerous studies showing—across different cardiovascular diseases and across countries—fewer hospitalizations, diagnostic and interventional procedures, outpatient consultations,^8^ and fewer emergency department visits took place during the pandemic, with those admitted being significantly younger and with a significantly shorter length of stay than before the pandemic.^6^ Results may be explained by limited access to public medical care, fear of infection, reorganization of medical services or lack of physical activity.^6^ Secondly, as for elective surgery, the pandemic worsened existing backlogs for elective procedures as resources for non-urgent care were diverted towards COVID-19 patients, and services were reduced in order to avoid other patients being infected.^9^ A recent Organization for Economic Cooperation and Development (OECD) report mentions differences in the effectiveness of containment measures, pre-existing capacity and the ability to mobilize additional resources as possible reasons for these indirect effects of the pandemic and the recovery of elective surgery interventions.^11^ Thirdly, results on trauma care are mixed. A study in the USA found different effects in the number of admissions depending on the type of trauma (e.g. an increase among older people due to falls), but a reduction in serious trauma admission rates, mortality and ICU admission rates.^12^ In the Netherlands, fewer patients sustained car- and sports-related injuries.^13^

Our study builds on the extant literature by identifying changes in selected indicators of health care utilization across Europe as a result of the COVID-19 pandemic in a systematic manner to inform service planning for future pandemics.

## Methods

### Federated analyses approach and aggregate local outputs

This research project has been carried out using the European Population Health Information Research Infrastructure’s (PHIRI) federated research infrastructure (FRI).^14^ PHIRI was developed to facilitate and generate the best available evidence for research on the health and well-being of populations impacted by COVID-19. See Phiri.eu^15^ and healthinformationportal.eu^16^ for more information. In essence, the PHIRI FRI is a network with a coordination node that orchestrates the workflow and the communication between various federated nodes that have access to sensitive health data. In this FRI, the coordination hub implements and containerizes the analytical pipeline in a stand-alone application that is deployed in each of the nodes. The nodes run the pipeline objects on the datasets prepared for the project and return the results to the orchestrating node for compilation and meta-analysis.

**Figure 1:**
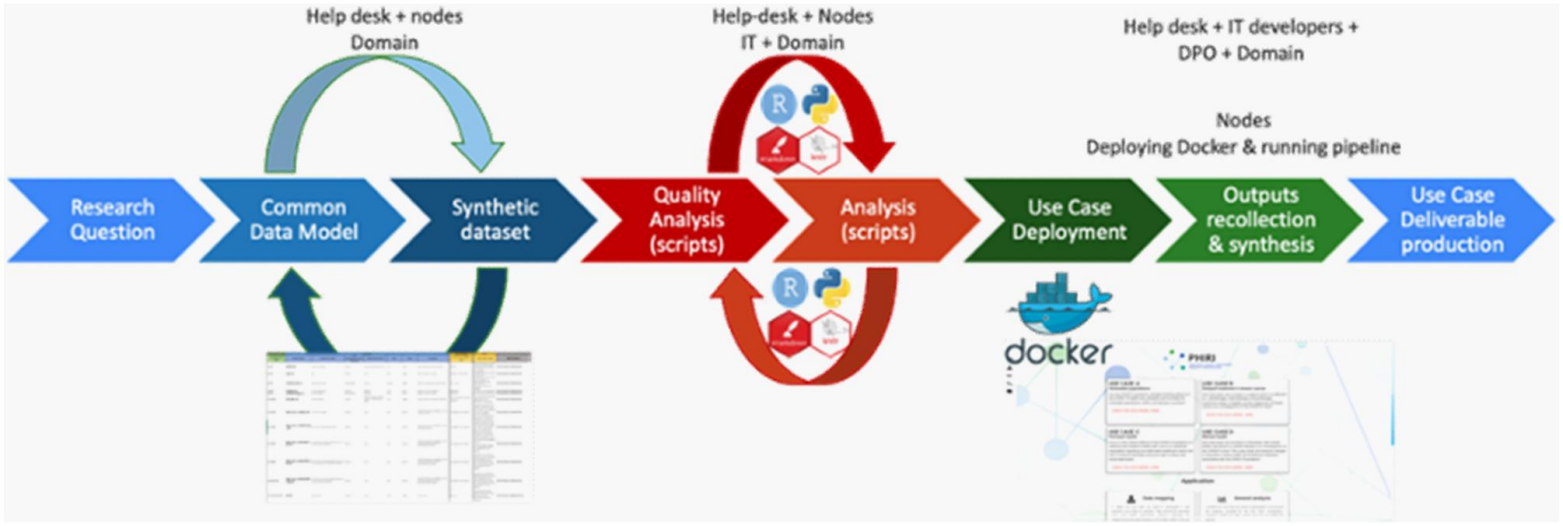
PHIRI standard workflow in the deployment of research questions

The workflow (figure 1) starts with the materialization of the research question in a data schema (i.e. information requirements) that becomes a common data model after some discussion rounds between the participating nodes. After the common data model is agreed, scripts for data quality assessment and algorithms for analysis are implemented upon a synthetic dataset prepared following the specifications of the data model. Finally, all the digital objects in the pipeline (i.e. common data model, synthetic dataset, data quality scripts and statistical algorithms) are containerized using Docker,^14^ and deployed in the different nodes’ premises, where researchers run the analyses and devolve the results.

The common data model specification supporting this paper can be found at https://zenodo.org/record/6357768 and the analytical pipeline can be found at https://zenodo.org/record/6377096.

### Study design

Our study examined patterns of three indicators of HCU across six regions (see table 1) between 1 January 2017 and 31 December 2021. Health care utilization can take several forms, and for the purpose of this paper, we investigated three forms of HCU derived from hospital admission data with different utilization pathways: (i) acute, urgent, non-elective diagnostic conditions mostly treated at the hospital that would be heavily impacted by the rapidity of a response; (ii) major, non-urgent, elective, chronic, procedure/intervention indicators that would usually be followed by a recovery period in hospital; (iii) non-fatal, acute, urgent, non-elective, diagnostic emergency care diagnoses that would ordinarily result in a hospital admission. This combination of forms of HCU were chosen to enable us to explore possible explanations for potential changes in HCU.

**Table 1:** A summary of the data contributed by each participating region

				Number of records provided				
Country/Region	Population at end of study period	Institution providing data	Coverage	Total	Heart attack	Stroke	Arthroplasty	Acute trauma
Aragon	1 326 261	Institute for Health Sciences in Aragon (IACS)	100% (Regional level)	38 406	5739	10 031	12 936	9700
Austria	8 932 664	Austrian National Public Health Institute (GOeG)	>99.5%	639 002	122 946	203 22	194 016	118 815
Croatia	3 871 833	Croatian Institute of Public Health (HZJZ)	100%	117 972	35 423	52 241	na^a^	30 308
Italy	59 236 213	Istituto Superiore di Sanità-ISS	100% (national data from hospital discharge records)	2 844 38	540 502	694 25	947 010	662 622
Netherlands^b^	17 403 108	Dutch National Institute for Public Health and the Environment (RIVM). Data source holders: Dutch Hospital Data and Vektis. Statistic Netherlands functioned as trusted third-party enabling linkage between datasets while ensuring the privacy of the involved individuals according to Dutch law (Statistics Netherlands act 2003)	100%	364 762	109 713	31 355	210 924	12 770
Wales	3 107 800	SAIL	86% GP coverage	110 194	28 080	28 080	31 901	22 133

a Not applicable.

b The study period of the Netherlands covered 2017–20, which was one year shorter than the other areas.

We defined events using the appropriate International Classification of Diseases (ICD), ICD-9 and 10 codes for these conditions and treatments codes which are used in the participating countries. Acute cardiovascular events were defined as vascular incidents restricted to acute myocardial infarctions (ICD-10 code I21) and stroke (ICD-10 codes I60–I64), and elective major joint replacement surgeries as total hip or knee replacements surgeries (OPCS codes W37-19 for hip replacements, W40-42 for knee replacements; ICD-9 codes 81.51–81.55, 00.70–00.73, 00.80–00.87). Our definition of serious trauma aims to capture serious but non-fatal injuries that result in a hospital admission using the injuries identified in Cryer et al.^10^ (ICD-10 codes S720–S723, S063, S272, S360–S361).

### Data sources

We invited all regions participating in PHIRI to contribute data towards this project. Participants had to be able to provide monthly level hospital admission data following the design of the common data model.^17^ Additionally all participating regions had to provide a summary of the available population as a suitable denominator for rate standardization.

Each provider constructed a regional cohort using individual-level linked, pseudonymized electronic health records (EHR) and administrative data sources available to the respective regions through their individual trusted research environments. The cohorts were assembled following a common data model provided at https://zenodo.org/record/6357768 as a template, before uploading to the use case A PHIRI FRI (found at https://zenodo.org/record/6936792 and https://zenodo.org/record/5729311). These harmonized data models allowed for a standardized methodology that could be applied to any participating regions and generate an aggregated data format suitable for an informative analysis or meta-analysis, while still abiding to local General Data Protection Regulation (GDPR) legislation.

The derived aggregate data produced by the PHIRI FRI from each participating organization’s common data model detailed the number of events of each type occurring in each month throughout the study period, subdivided by age group (10-year banding) and sex. Each region was also requested to compile a summary of the population available to them, also stratified by age and sex. These data were exported from their respective nodes.

### Statistical analysis

The aggregated HCU data and population summary data were combined with the 2013 European standard population^18^ to calculate a European age-standardized rate (EASR) and European age-standardized counts for each region (EASC) using Equation 1. The EASRs and 95% confidence intervals (95% CI) were calculated for each region following Office for National Statistics (ONS) guidelines^19^ for a visual comparison. This standardized count allows for a meaningful cross-regional comparison of rates of HCU.

Equation 1: The equation used to calculate EASR. *p* = population, *n* = number of events, esp = European standard proportion, *i* = age group:


(1) $$\mathrm{easr}=\sum \left(\frac{n_{i}}{p_{i}}\times 100 000\right)\times esp_{i}\;$$

Equation 2: The equation used to calculate EASC from EASR. *p* = population:


(2) $$\mathrm{easc}=\;\frac{\mathrm{easr}}{100\;000}\times p$$

We used forecast modelling to identify changes from predicted EASCs of HCU for each region individually. The analysis was carried out separately for males and females. We fitted negative-binomial regression models accounting for seasonal variation^20^ using Fourier terms to 36 months of HCU data (January 2017 to December 2019). These models were used to predict the expected HCU and associated 95% prediction intervals for January 2020 to December 2021. The European age-standardized observed data from January 2020 to December 2021 were plotted against this forecasted HCU to observe any deviation from the expected range of values.

Data aggregation, analyses and data visualization were performed in RStudio (v4.1.3). The forecasting was performed using the *Trending* package.^21^

### Ethics and permissions

Each data hub retrieved the necessary approvals to meet their relevant Ethic Committee requirements. All the GDPR and Helsinki declaration prescriptions (at that level) are responsibility of the data hubs (national nodes or data holders). Individual written patient consent was not required for this study.

## Results

Data were provided by six regions in Europe in aggregate form (table 1). Aragon, Austria, Croatia, Italy and Wales were able to provide data for the full study period, and The Netherlands were able to provide data from study start until 31 December 2020. The Netherlands experienced a limitation due to potential disclosure risk as a result of small numbers, and instead of providing aggregated data, performed their EASR and EASC calculations within their own trusted research environment and contributed these data. All regions were able to provide data regarding acute cardiovascular events and serious trauma, and all but Croatia were able to provide information detailing elective surgeries.

### European age standardized rates

**Figure 2:**
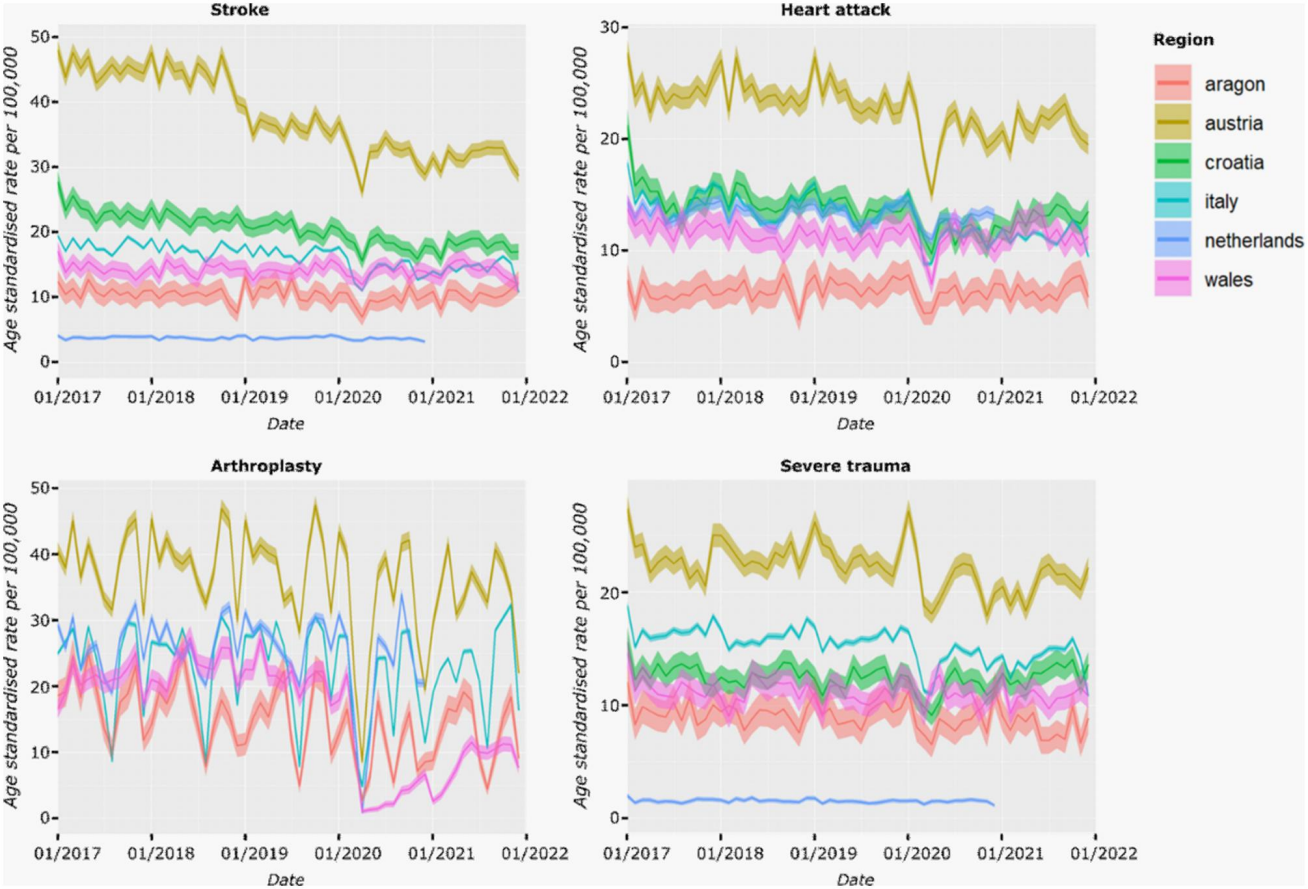
European age-standardized rates (solid lines) and the 95% CI (shaded area) across four selected forms of HCU in six regions in Europe

We found that rates of HCU varied by region, but most had a visible decline in rates in February and March 2020 (figure 2).

### Forecasting

Forecasting results revealed differences in the number of age-standardized events following the onset of the pandemic between region, sex and HCU.

Cases of stroke showed a decrease in incidences below the 95% prediction interval immediately following March 2020 in Austria, Italy, Welsh females and Aragon males (Supplementary figure S1). All regions except Italy immediately returned to pre-pandemic counts; however, Austrian and Welsh females recorded fewer-than-expected incidents at the end of 2020. Italy experienced fewer incidents than the forecasted range for February to April 2020, and returned to the predicted range during the summer before falling to lower-than-expected for several months in winter 2020/2021. The Netherlands showed no significant change immediately following the pandemic; however, they reported fewer-than-expected cases at the end of 2021. Croatia, Aragon females and Welsh males showed no change in incidents of stroke outside of the forecasted range following the start of the pandemic in the observed period.

**Figure 3:**
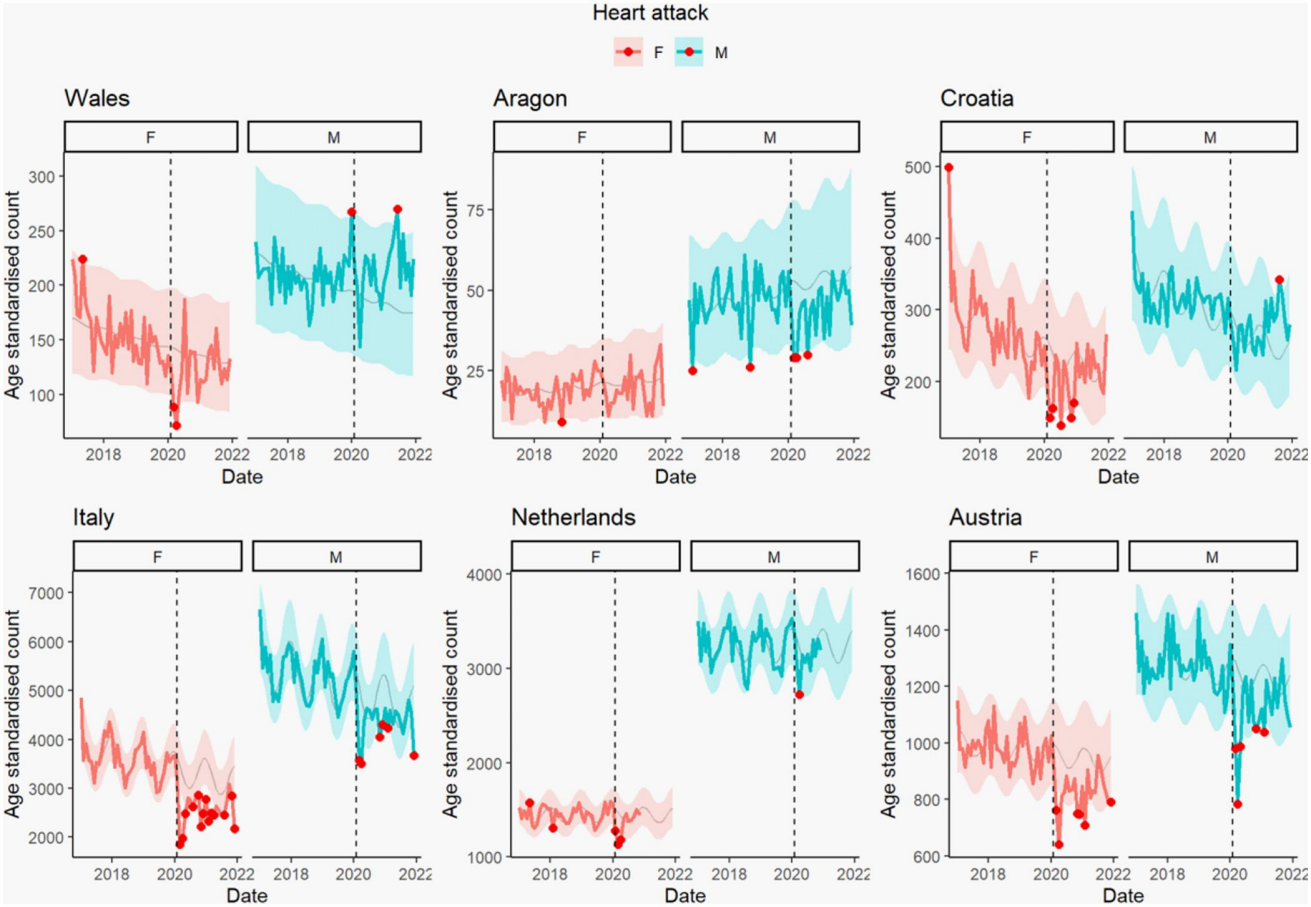
The forecasting results for heart attack in six regions of Europe. The dotted line indicates the start of 2020. The solid line indicates real data and the highlighted region is the 95% prediction intervals, representing the expected range of values generated by the model. All data before the dotted line were used to create the forecast model, and the red dots highlight data that deviated from the expected range

Every region included in the forecasting model showed some deviation from predicted incidents of heart attack (figure 3). Austria had the largest relative decrease in both males and females in the early stages of the pandemic. Heart attack admissions in Austria returned to the range of prediction within 2 months, but also experienced fewer-than-expected incidents the following winter ending in 2021 and additionally for females in the winter ending in 2022. Italian males experienced fewer-than-expected incidents during the initial COVID-19 wave, and the following winters ending in 2021 and 2022, while Italian females experienced a more severe relative decrease in cases overall, that did not return to pre-pandemic levels by study end. The Dutch, Croatian and Welsh females as well as Aragon males all fell to below expected counts immediately following March 2020, but quickly returned to pre-pandemic levels, except for Croatian females who continued to experience fewer-than-predicted cases of heart attack hospital admissions during the rest of 2020.

In all the considered regions, arthroplasty was the most affected by the COVID-19 pandemic. Arthroplasty incidents fell to below predicted levels in all regions in both males and females following the onset of the pandemic (Supplementary figure S2). Austria, The Netherlands and Wales had the largest relative decreases in arthroplasties following March 2020, with Austria and The Netherlands also experiencing lower-than-expected surgeries towards the end of 2020 (the Netherlands and Austria) and 2021 (Austria only). Austria and the Netherlands quickly returned to pre-pandemic levels regarding arthroplasties following the initial wave. By contrast, Wales experienced a gradual increase in surgeries after the initial drop but did not return to pre-pandemic levels by the conclusion of the study period. In both Aragon and Italy, the COVID-19 pandemic effect was lower in comparison to the other regions, with a slight decrease in arthroplasty surgeries for 2–3 months following the initial wave before returning to pre-pandemic levels.

Serious trauma was the least impacted by the COVID-19 pandemic. Italy and Austria reported fewer-than-expected cases immediately following the pandemic, and again at the end of 2020. Italy also experienced fewer-than-expected cases at the end of 2021. Aragon females and Croatian males also experienced fewer-than-expected counts immediately following the pandemic but returned to the expected range within one or two months. Results for the Netherlands, Wales, Aragon males and Croatian females did not show any significant change in hospital admissions for serious trauma.

## Discussion

Our comparative analysis highlights a great deal of variation between participating regions of Europe regarding selected forms of healthcare utilization following the COVID-19 pandemic. We found that elective surgeries were most affected, with arthroplasties falling below predicted levels in all regions for both male and female populations. Cardiovascular events were the second most affected by the pandemic among the conditions included. We found lower-than-expected cases in every region for heart attacks and stroke (except for Croatia and the Netherlands in the latter case). In addition, these two conditions displayed some differences between males and females that could not be explained by our study design. Further research is needed to better understand the relevance of sex and/or gender patterns for these types of acute crisis events, and how they may be impacted differently by the pandemic.^22^ Finally, acute traumas were consistently lower-than-expected in Italy and Austria but remained within expectations for all other regions.

Our methodology captures a range of forms of HCU, each of which will have had a distinct relationship with HCU during the pandemic. The causes for these decreases in HCU for acute cardiovascular events remain unclear and will require further investigation to determine whether these are the result of changes in occurrence or reporting. A decrease in recordings of cardiovascular events could, for example, be the result of minor events such as non-ST elevation myocardial infarction (NSTEMI) or a transient ischaemic attack (TIA) going unreported. Alternatively, they could be the result of an increase in deaths at home that are not making it into hospital admissions data.^23^ The decrease in acute cardiovascular HCU could also be the results of a harvesting or substitution effect. The high mortality rates at the beginning of the pandemic could be responsible for the subsequent fall in these HCU, additionally many people who were at risk of acute cardiovascular HCU would have been high risk for COVID-19 mortality too.^24^ However, if this was the sole cause, we would expect to see a larger decrease in males who were at a higher risk of COVID-19 mortality,^25^ which is the opposite to our observations. A more definitive answer to whether a decrease in cardiovascular events is a result of a decrease in incidents or a decrease in reporting will be of great importance to future work.

The observed decrease in elective surgeries can be attributed to a streamlining of resources towards urgent medical care and away from major elective procedures.^26^ The speed of recovery for elective arthroplasty in many regions provides insight into the resilience of the health systems in place, which will be of interest to health planners. Wales shows particularly poor recovery regarding this form of HCU, but may be due in part to policy decisions to repurpose operating theatres as emergency care units.^27^ However, these adjustments were no longer in place by the end of our study period. There is evidence in the UK that self-funded hip and knee replacements have increased by 165% and 122%, respectively, when comparing July to September 2019 figures against the same period in 2021.^28^ These outcomes would not be detected in the Welsh data collection and may partially account for the prolonged lower-than-expected arthroplasties for Wales; however, further investigation is needed to quantify the effect of this. These results indicate that there will likely be a backlog of postponed operations across all regions which may be being alleviated by privately funded arthroplasties.

For serious trauma, we found mixed results across regions in relation to the pandemic, with clear decreases in occurrences in Italy and Austria, and the remainder being largely unaffected. The selected serious traumas are unlikely to go unreported and should reflect the true numbers of incidents occurring in the region. This is in line with published literature, showing that the impact of COVID-19 on trauma HCU is mixed.^3^ The types of events we selected are most likely to be the results of road traffic collisions and low impact falls (falls from standing). Road traffic collisions were shown to fall across Europe,^29–31^ with the most significant reduction in traffic volume and road death occurring in Italy. However, traffic volume and road-related deaths fell more in Croatia, Spain and the UK than they did in Austria, which is not reflected in our results. Research into low impact falls in Europe is mixed regarding changes in prevalence resulting from the pandemic.^30,^^32–34^

A key finding from our results is the indication of pandemic-related changes in acute cardiovascular associated HCU disproportionately affecting women. Previous research has highlighted that rates of TIA and heart attack misdiagnosis are higher in females compared with males and that females are less likely to report stroke symptoms.^35–38^ In a diagnostic area where women are already being misdiagnosed and experience referral delays, possibly also due to gendered patterns in diagnosis and care, a proportionally larger decrease in cases as results of the pandemic should be particularly concerning to health care providers.

The strength of this study comes from the use of the PHIRI FRI system, allowing multiple regions in Europe to participate with individual-level health data in our study in a standardized way, despite the variety of reporting systems in place across these locations. This research structure is highly suited to replication and is scalable to include data from additional regions and timeframes. However, not all data were available or linkable in all regions for the full study period; these regions will not be able to as thoroughly evaluate the impact of the pandemic on their health system.

In this paper, we used a generalized linear modelling (GLM) based approach accounting for seasonality to compare estimated and observed healthcare utilization. Other modelling approaches such as auto regressive integrated moving average (ARIMA) models with exogenous variables (ARIMAX) could also be used.^39^ The main advantage of a GLM-based approach over auto-regressive-based approaches in this setting are that they describe covariate effects and negative correlations in a straight forward way, increasing interpretability of the findings. Further work should be undertaken to combine these data into a cross-regional statistical method that can identify demographic and epidemiological influences on HCU across these regions as a whole. Additionally, the inclusion of region specific COVID-19 mitigation measures, healthcare factors such as hospital bed provision or the availability of vaccines could provide valuable insight into patterns of HCU in these regions.

Further work should also be undertaken to identify the mechanisms involved in a reduction of HCU. A better understanding of whether these decreases are a result of decreased incidences, decreased healthcare-seeking behaviour, or other causes, is needed. This can also be applied to demographic disparities, identifying the underlying mechanisms responsible for differences in HCU between the sexes. Also, linking the dynamics of HCU with outcomes research would be very valuable to approach a more complete picture of effects on health systems and population health.

We found that the COVID-19 pandemic has had an impact on our selected indicators of HCU across the participating regions of Europe, with some notable differences in the speed of recovery in different jurisdictions. Further research needs to be undertaken to identify more specific factors associated with the change in health care utilization, including regional COVID-19 factors, system differences in health care provision and organization and individual-level factors.

## Supplementary Material

ckad180_Supplementary_Data

## Data Availability

These results correspond to the international comparison analysis for countries participating in PHIRI WP6 Use Case A investigating changes to existing healthcare utilization as indirect effect of the COVID-19 pandemic. The aggregation and analytical scripts, in addition to the PHIRI FRI are available at https://doi.org/10.5281/zenodo.5729310. The patient-level and in some cases the aggregate-level data underlying this article provided by all participating regions cannot be shared publicly due to data protection and confidentiality requirements. European age-standardized rates and counts can be made available to approved researchers after securing relevant permissions from the data holders via the relevant approval pathways. Key points The COVID-19 pandemic has impacted healthcare utilization across Europe. We find considerable variation between regions, forms of health care utilization and the extent to which health care utilization was affected by the pandemic and its recovery. We find evidence of a disparity between sexes and their healthcare utilization during the pandemic. The PHIRI infrastructure provides a mechanism for rapid analysis of between country and region differences to assess the indirect impacts of events such as pandemics

## Notes

On behalf of the Population Health Information Research Infrastructure
