# Supplementary material for: Has the COVID-19 pandemic changed existing patterns of non-COVID-19 health care utilization? A retrospective analysis of six regions in Europe
Source: Eur J Public Health. 2024 Jul 1;34(Suppl 1):i67–73. doi: 10.1093/eurpub/ckad180 (PMC11631558; doi:10.1093/eurpub/ckad180)
Supplement: ckad180_Supplementary_Data [file ckad180_supplementary_data.pdf]

Supplementary:

Table S1 – Diagnostic codes used to identify individuals who correspond to our definition of acute cardiovascular event, elective surgery or severe trauma

**Acute cardiovascular event**	**ICD-10 code**
Myocardial infarction	I21
Subarachnoid haemorrhage	I60
Intracerebral haemorrhage	I61
Other nontraumatic intracranial haemorrhage	I62
Cerebral infarction	I63
Stroke, not specified as haemorrhage or infarction	I64

**Elective surgery**	**OPCS codes**
Total hip replacement with acetabular bone graft	W37-39
Primary total prosthetic replacement of knee	W40-42

**Elective surgery**	SDC codes
Specialty hip replacement	305
Arthrosis pelvis/hip/femur	1701
Care product (hip replacement)	131999052 or 131999051
Care activity (hip replacement)	38567
Specialty knee replacement	305
Arthrosis knee	1801
Care product (knee replacement)	131999103 or 131999104
Care activity (knee replacement)	38663

**Severe trauma**	**ICD-10 code**
Fracture to head or neck of femur	S720
Displaced subtrochanteric fracture of right femur	S721
Subtrochanteric fracture of femur	S722
Fracture of shaft of femur	S723
Focal traumatic brain injury	S063
Traumatic hemopneumothorax	S272
Injury of spleen	S360
Injury of liver and gallbladder and bile duct	S361

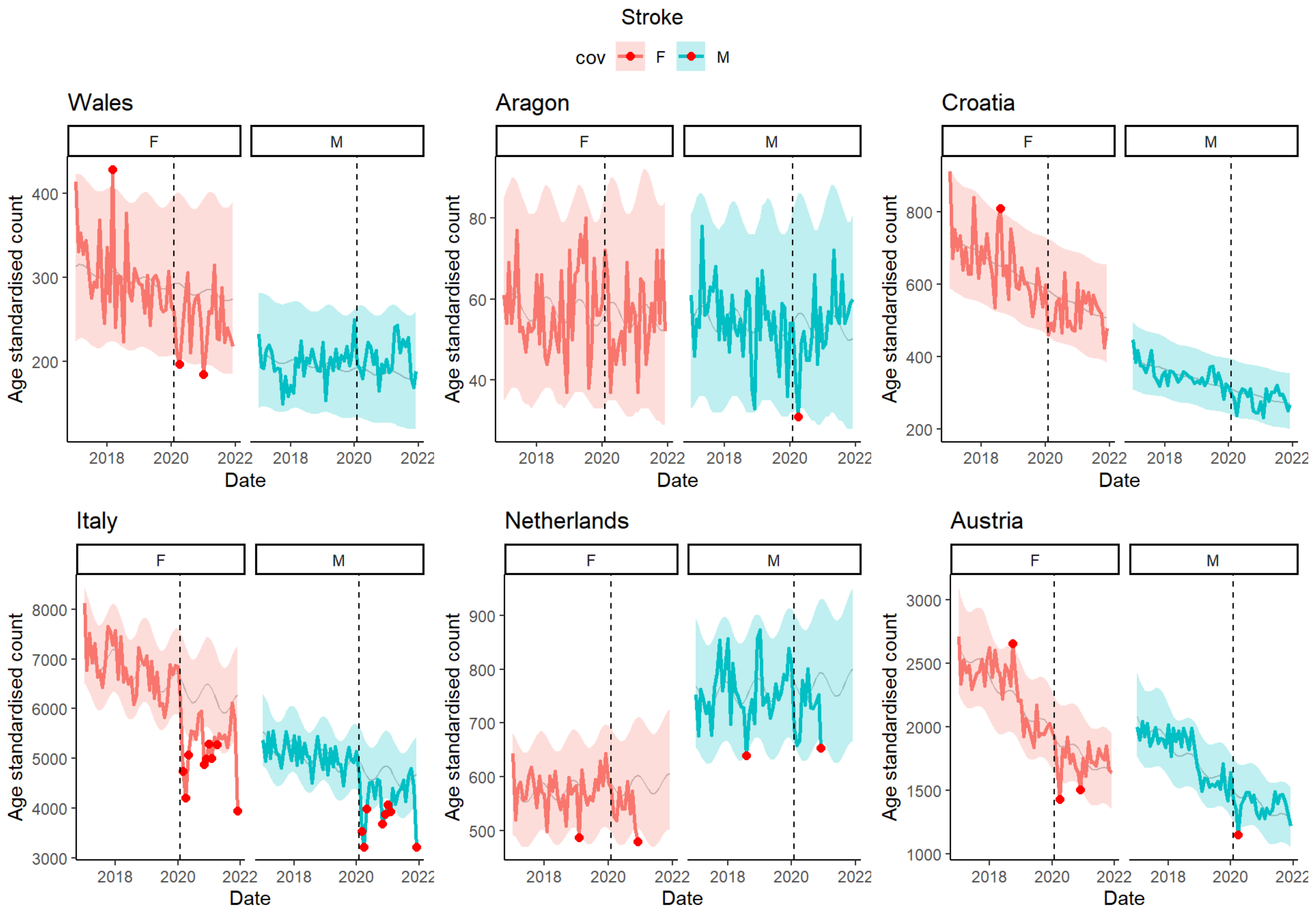

Supplementary Figure 1 The forecasting results for stroke in six regions of Europe. The dotted line indicates the start of 2020. The solid line indicates real data and the highlighted region is the 95% prediction intervals, representing the expected range of values generated by the model. All data before the dotted line was used to create the forecast model, and the red dots highlight data that deviated from the expected range

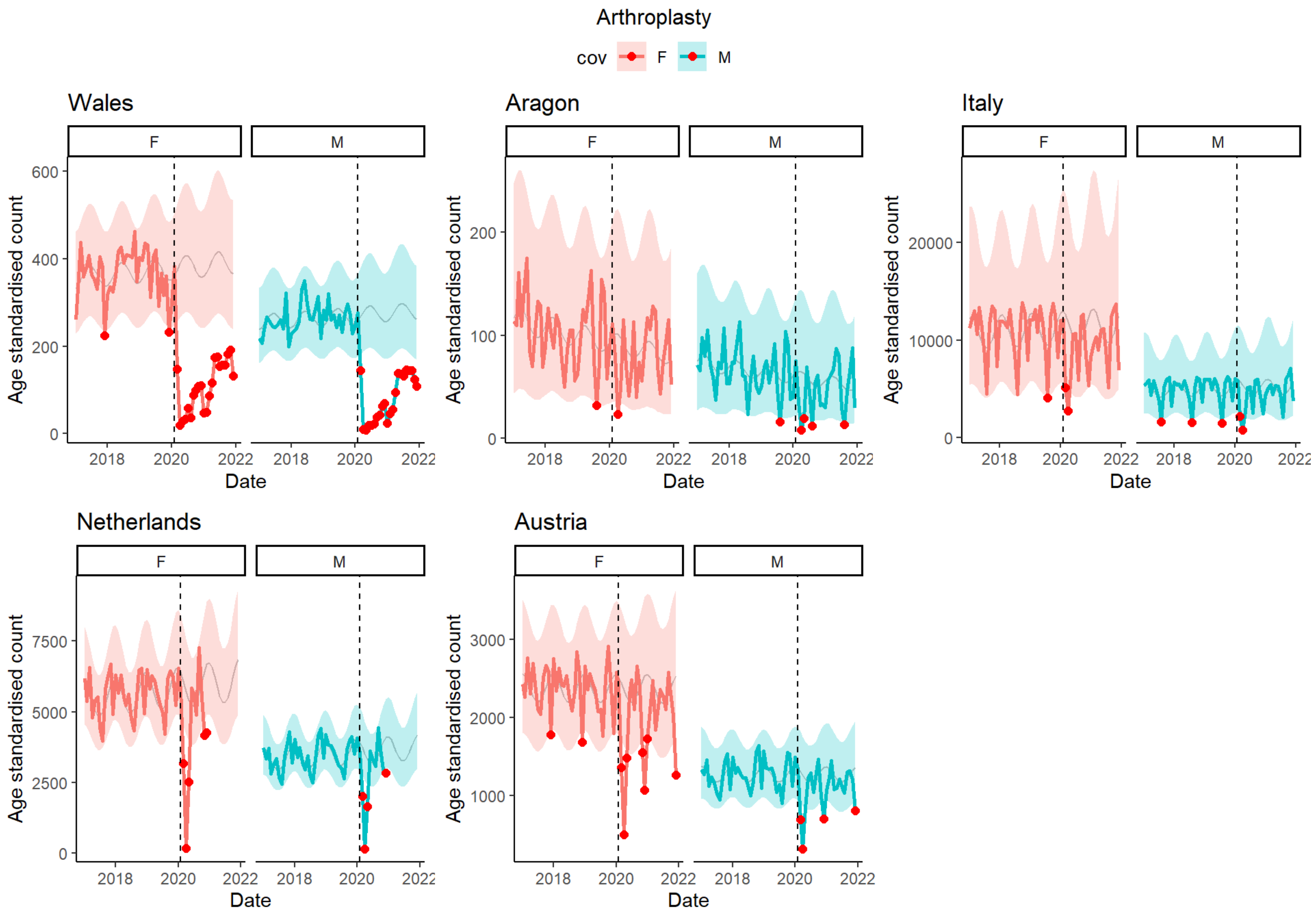

Supplementary Figure 2 The forecasting results arthroplasty in five regions of Europe. The dotted line indicates the start of 2020. The solid line indicates real data and the highlighted region is the 95% prediction intervals, representing the expected range of values generated by the model. All data before the dotted line was used to create the forecast model, and the red dots highlight data that deviated from the expected range

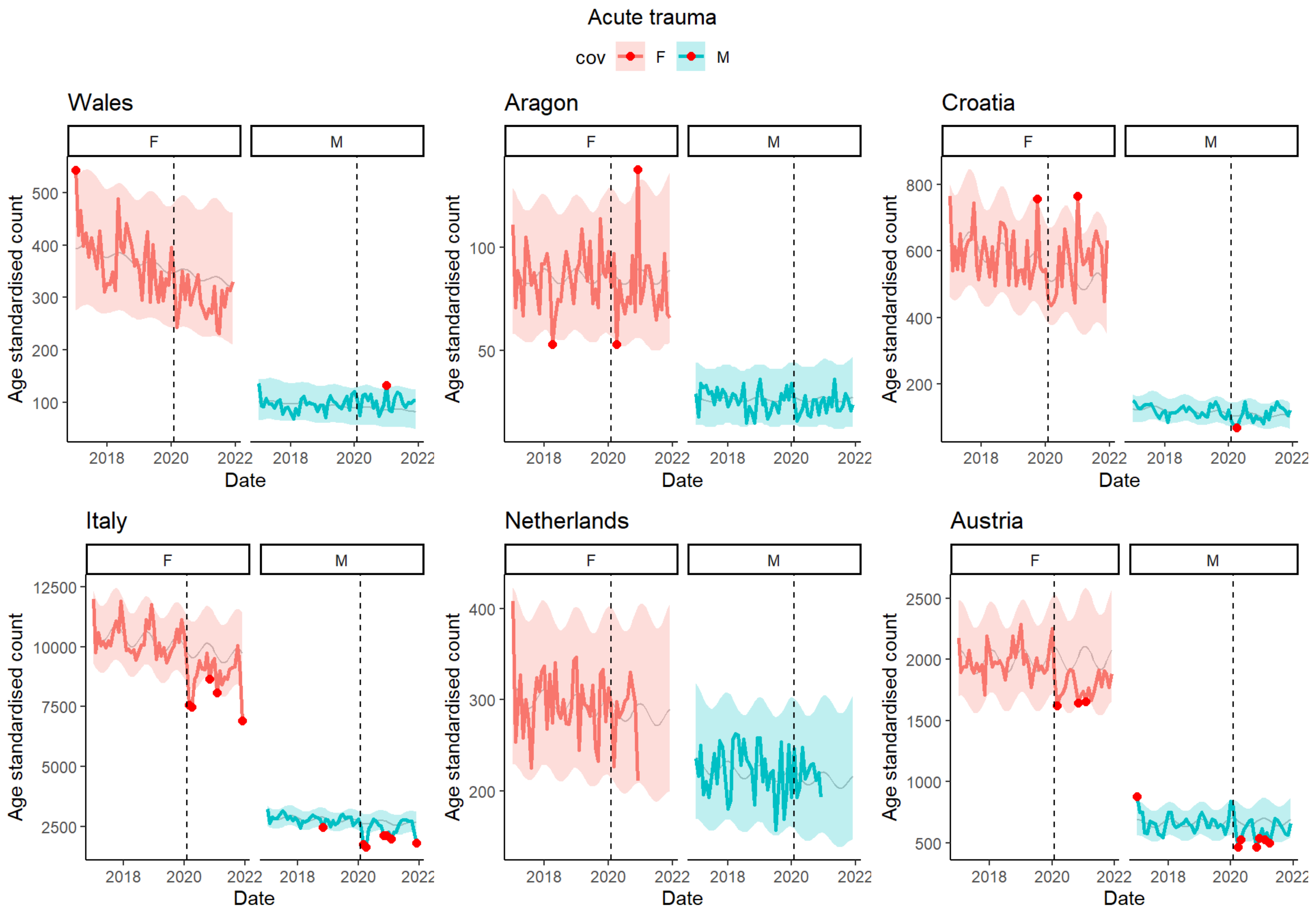

Supplementary Figure 3 The forecasting results for Severe trauma in six regions of Europe. The dotted line indicates the start of 2020. The solid line indicates real data and the highlighted region is the 95% prediction intervals, representing the expected range of values generated by the model. All data before the dotted line was used to create the forecast model, and the red dots highlight data that deviated from the expected range
